# Stable SNP Allele Associations With High Grain Zinc Content in Polished Rice (*Oryza sativa* L.) Identified Based on ddRAD Sequencing

**DOI:** 10.3389/fgene.2020.00763

**Published:** 2020-08-11

**Authors:** P. Madhu Babu, C. N. Neeraja, Santosha Rathod, K. Suman, G. Anurag Uttam, Navajeet Chakravartty, V. B. Reddy Lachagari, U. Chaitanya, Lella V. Subba Rao, Sitapati Rao Voleti

**Affiliations:** ^1^ICAR-Indian Institute of Rice Research, Hyderabad, India; ^2^AgriGenome Labs Pvt. Ltd., Hyderabad, India

**Keywords:** rice, grain zinc, stable donors, ddRAD sequencing, SNP, candidate genes

## Abstract

Polished rice is widely consumed staple food across the globe, however, it contains limited nutrients especially iron (Fe) and zinc (Zn). To identify promising genotypes for grain Zn, a total of 40 genotypes consisting 20 rice landraces, and 20 released high yielding rice varieties were evaluated in three environments (wet seasons 2014, 2015 and 2016) for nine traits including days to 50% flowering (DFF), plant height (PH), panicle length (PL), total number of tillers (TNT), single plant yield (SPY), Fe and Zn in brown (IBR, ZBR) and polished rice (IPR, ZPR). Additive Main Effect and Multiplicative Interaction (AMMI), Genotype and Genotype × Environment Interaction (GGE) analyses identified genotypes G22 (Edavankudi Pokkali), G17 (Taraori Basmati), G27 (Chittimuthyalu) and G26 (Kalanamak) stable for ZPR and G8 (Savitri) stable for SPY across three environments. Significant negative correlation between yield and grain Zn was reaffirmed. Regression analysis indicated the contribution of traits toward ZPR and SPY and also desirable level of grain Zn in brown rice. A total of 39,137 polymorphic single nucleotide polymorphisms (SNPs) were obtained through double digest restriction site associated DNA (dd-RAD) sequencing of 40 genotypes. Association analyses with nine phenotypic traits revealed 188 stable SNPs with six traits across three environments. ZPR was associated with SNPs located in three putative candidate genes (LOC_Os03g47980, LOC_Os07g47950 and LOC_Os07g48050) on chromosomes 3 and 7. The genomic region of chromosome 7 co localized with reported genomic regions (rMQTL_7_._1_) and OsNAS3 candidate gene. SPY was found to be associated with 12 stable SNPs located in 11 putative candidate genes on chromosome 1, 6, and 12. Characterization of rice landraces and varieties in terms of stability for their grain Zn and yield identified promising donors and recipients along with genomic regions in the present study to be deployed rice Zn biofortification breeding program.

## Introduction

Biofortification is one of the promising strategies to address malnutrition through enhancing the nutritional value of crops and more than 200 biofortified crop varieties have been released worldwide (HarvestPlus and FAO, 2019). Rice, being major staple food for half of the world population has been targeted for biofortification for various nutrients since 2000 using genetic engineering and conventional breeding approaches (Mahender et al., 2016; Neeraja et al., 2017; Majumder et al., 2019; Ludwig and Slamet-Loedin, 2019). Zinc (Zn) is one of the most essential nutrients for human health and associated with various metabolic activities (Roohani et al., 2013). Enhancing of Zn content of the rice grains could have a positive impact on human health (Graham et al., 2001; Stein, 2010). Polished rice, the most preferred form for consumption is poor source of Zn with a range of 8 to 12 ppm. Popular rice varieties usually contain lesser micronutrients in grains compared to the traditional cultivars and landraces (Nachimuthu et al., 2015; Pradhan et al., 2020; Rao et al., 2020). Through conventional breeding, enhancement of grain Zn without yield compromise in polished rice has been demonstrated and many rice varieties with increased Zn content have been released in a few Asian countries *viz*., 10 in India, three in Bangladesh, one each in Indonesia and Philippines^1,2^ (Palanog et al., 2019). A range of grain Zn from 20 to 28 ppm in polished rice and similar or higher yield levels as popular adopted varieties along with standard quality traits are mandatory for the released of Zn biofortified rice varieties as defined by the government agencies in different countries. Thus, for the development of biofortified rice varieties, high grain Zn content, yield and quality should be combined. For developing such varieties, availability of suitable germplasm is the foremost requirement. Wide genetic variability was reported for grain Zn ranging from in brown (7.3 to 52.7 ppm) and polished (8 to 38 ppm) rice and several donors have been identified (Swamy et al., 2016; Rao et al., 2020). Traditional varieties or landraces are known to be the source of novel genes/alleles for the agronomically important traits including grain Zn content (Neeraja et al., 2018; Bollinedi et al., 2020). Increasing grain Zn content in rice was reported to be feasible by utilizing germplasm in the breeding programs to reach the international standards of grain Zn 28 ppm in polished rice (Rao et al., 2020^1^).

The success of genetic improvement programs depends on the selection of productive and stable genotypes which depends on understanding of the interaction between genotypes and environments (G × E). Breeding for high grain Zn is slow because of low heritability and genetic interactions such as epistasis, environmental-genotype interactions, and polygenic effects (Zhang et al., 2014; Pradhan et al., 2020). To study G × E interactions, multivariate techniques such as biplots; Additive Main effects and Multiplicative Interaction (AMMI) and Genotype main effects and G x E interaction effects (GGE) are being widely adopted (Gauch and Zobel, 1997; Li et al., 2017). Considering the influence of environment on performance of genotypes for high grain Zn, effects of genotype (G), environment (E) and G x E were studied through analysis of variance (ANOVA), AMMI and GGE model in rice (Malosetti et al., 2013; Inabangan-Asilo et al., 2019).

Identification of candidate genes/genomic regions controlling high grain Zn would be an important approach for the marker assisted breeding of biofortified rice varieties. Using bi-parental mapping populations, 22 independent studies have reported 220 QTL for grain Fe and Zn in rice using simple sequence repeat (SSR) markers or candidate gene based markers (Raza et al., 2019). Either a major QTL > 30% phenotypic variance (PV) or pyramiding of a few minor QTL (∼20% PV) can be deployed in the marker assisted breeding of rice Zn biofortified rice varieties. Association mapping of grain Fe and Zn was also studied using SSR and candidate gene based markers in rice (Pradhan et al., 2020). Using next generation sequencing (NGS) approaches, genome-wide single-nucleotide polymorphic (SNP) molecular markers can be generated for the efficient discovery of the genomic regions associated with complex traits. A modified NGS approach based for scoring of SNPs based reduced representation of genome is known as genotyping-by-sequencing (GBS) (Deschamps et al., 2012) and is being applied in mapping of QTL and identification of genes in rice (De Leon et al., 2016; Furuta et al., 2017). Restriction-site associated DNA sequencing (RAD-seq) involves restriction enzyme digestion and NGS of regions adjacent to restriction sites, results in high throughput genetic markers across the genome (Baird et al., 2008; Davey et al., 2011; Peterson et al., 2012). A modified RAD-seq based on two restriction enzymes comprising a rare-cutting and frequently cutting as double-digest RAD-seq (ddRAD-seq) increases the selection of stable and repeatable size regions across samples (Peterson et al., 2012) and has been deployed in several crop plant species (Bodanapu et al., 2019; Gali et al., 2019; Sudan et al., 2019; Tafesse et al., 2020). Recently, SNPs associated with Fe, Zn and selenium (Se) concentration in field pea (*Pisum sativum* L.) were identified using dd RAD-seq in field pea (Dissanayaka et al., under early view). Using SNP markers, genomic regions for grain Zn along with other minerals have also been identified in rice using genome wide association studies (GWAS) (Norton et al., 2010; Zhang et al., 2014; Pradhan et al., 2020; Bollinedi et al., 2020) and also in Multi-parent Advanced Generation Inter-Cross (MAGIC) population (Descalsota et al., 2018).

The objectives of the present study were to identify stable donors for high grain Zn from shortlisted promising landraces in comparison to popular high yielding varieties based on evaluation for three years; correlate high grain Zn with yield and other traits; and identify polymorphic genome wide SNPs and their associations with high grain Zn, yield and other traits in the studied genotypes.

## Materials and Methods

### Plant Material

In our earlier studies during 2013, > 5000 rice genotypes consisting landraces from various parts of India, breeding lines and high yielding varieties were evaluated for their grain Zn content (Babu et al., 2014). A few promising landraces and high yielding were shortlisted based on their wide variation of grain yield and zinc content (Table 1). The set of 40 genotypes with contrasting grain Zn and yield were evaluated for their agro-morphological and yield traits along with grain Fe and Zn content in brown and polished rice over a period of three wet seasons of 2014, 2015, and 2016.

**TABLE 1:** Details of pedigree for released rice varieties and origin for rice landraces in the study.

**S No**	**Code**	**Variety Name**	**Designation**	Cross Combination/origin*
1	G 01	Samba Mahsuri	BPT 5204	GEB 24/TN1//Mahsuri
2	G 02	Vijetha	MTU 1001	MTU 5249/MTU 7014
3	G 03	Cottondora Sannalu	MTU 1010	Krishnaveni/IR 64
4	G 04	Swarna	MTU 7029	Vashistha/Mahsuri
5	G 05	IR 64	IR 18348-36-3-3	IR 5657-33-2-1/IR 2061-465-1-5-5
6	G 06	PR 116	PAU 2020-10-3-1	PR 108/PAU 1628//PR 108
7	G 07	Narendradhan 359	NDR 359	BG 90-2-4/OB 677
8	G 08	Savitri	CR 210-1009	Pankaj/Jagannath
9	G 09	Pusa Basmati 1	Pusa 615-140-10-1	Pusa 167/Karnal local
10	G 10	Jaya	12306	TN1/T141
11	G 11	Mahsuri	-	Taichung 65/Mayang Ebos 6080/2
12	G 12	Lalat	ORS26-2014-4	OBS677/IR2071//Vikram/W1263
13	G 13	Sampada	PR4-60-105-6-8-2-5-1B	Vijaya/C14-8
14	G 14	Pushyami	MTU 1075	MTU 2716/MTU 1010
15	G 15	Jalpriya	NDGR 150	IET 4060/Jalmagna
16	G 16	ARB-45	Azucena/Moromutant	Breeding line
17	G 17	Taraori Basmati	HBC 19	Pure line selection from HBC 19
18	G 18	Akut Phou	KD 14-1-9	Lang Phou/IR 1364-37-3-1
19	G 19	Seetasail	Landrace	North India
20	G 20	Tilakkachari	Landrace	North India
21	G 21	High Iron Rice	Landrace	South India
22	G 22	Edavankudi Pokkali	Landrace	South India
23	G 23	Kadamakudy Pokkali	Landrace	South India
24	G 24	DRRDhan 45	IR80463−B−39−3	Areumbyeo/IRRI123
25	G 25	HP-5	IR80463−B−39−3-1	AreumbyeO/IRRI123
26	G 26	Kalanamak	Landrace	North India
27	G 27	Chittimuthyalu	Landrace	South India
28	G 28	Kala Jira Jaha	Landrace	North East Collection
29	G 29	Raga-binni	Landrace	North East Collection
30	G 30	Gopalbhok	Landrace	North East Collection
31	G 31	Hatibandha	Landrace	North East Collection
32	G 32	Mima	Landrace	North East Collection
33	G 33	Jahagipok	Landrace	North East Collection
34	G 34	Jahagisim	Landrace	North East Collection
35	G 35	Kewelhi lolu-R I	Landrace	North East Collection
36	G 36	Nedu	Landrace	North East Collection
37	G 37	Sirarakhong Manui	Landrace	North East Collection
38	G 38	Wungrei	Landrace	North East Collection
39	G 39	Arunachal pradesh-1	Landrace	North East Collection
40	G 40	Sakha	Landrace	North East Collection

### Field Experimental Details

Field experiments were conducted at research farm, Indian Council of Agricultural Research (ICAR)-Indian Institute of Rice Research (IIRR) (17°19′N and 78°29′E), Hyderabad, Telangana State, India during three consecutive wet seasons (*Kharif*) considered as three environments *viz*., 2014 (E1), 2015 (E2) and 2016 (E3) (Supplementary Table S1). The range of experimental soil characteristics across three years were: pH 8.2 – 8.4; non-saline (EC 0.7l – 0.72 dS/m); calcareous (free CaCO_3_ 5.01 – 5.04%); CEC 44.1 – 45.2 C mol (p +)/kg soil and medium soil organic carbon (0.69 – 0.72%); low soil available nitrogen (228 – 230 kg ha^–1^); high available phosphorus (105 –108 kg P_2_O_5_ ha^–1^), high available potassium (530 – 540 kg K_2_O ha^–1^), and high available Zn (12.5 –14.0 ppm). One month old seedlings of 40 genotypes were transplanted in a randomized complete block design with three replications. In each replication, every genotype was grown in one m^2^ plot (33.3 plants) with 20 cm row spacing and 15 cm intra-row spacing. Recommended package of rice crop production and protection practices were followed^3^.

### Trait Measurements

The genotypes were evaluated for days to 50% flowering (DFF), plant height (PH) (cm), number of tillers per plant (TNT), panicle length (PL) (cm), single plant yield (SPY) (g), grain Fe and Zn content in brown and polished rice (IBR, ZBR, IPR, ZPR) (ppm). The observations were recorded for three representative uniform plants from the center of the plot of each genotype. The seeds of each genotype and replication were dehusked using JLGJ4.5 testing rice husker (Jingjian Huayuan International Trade Co., Ltd.) and polisher (Krishi International India Ltd.) with non-ferrous and non-zinc components. Each sample of brown and polished rice (5 g) was subjected to energy dispersive X-ray fluorescent spectrophotometer (ED-XRF) (OXFORD Instruments X-Supreme 8000) at ICAR-IIRR as per standardized protocols (Rao et al., 2014).

### Statistical Analysis

Descriptive statistics such as mean, standard error of mean (SEm), skewness, kurtosis and coefficient variations (%) were calculated to understand the characteristics, dispersion and heterogeneity of traits of the study. Graphical representation of summary statistics was depicted in frequency distribution plots and boxplots using R software (R Core Team, 2018). ANOVA was used to compare the variation in agro-morphological and yield traits along with grain Zn and Fe content among 40 genotypes across three years and G × E interactions of 40 genotypes. The performance of genotypes was assessed using stability models *viz*, (1) Additive Main effects and Multiplicative Interaction (AMMI) (Gauch and Zobel, 1997), and (2) GGE Biplot or Site Regression model (Yan and Kang, 2002).

The AMMI model is a combination of ANOVA and principal component analysis (PCA), where the additive (main) effects were estimated using ANOVA and G × E interaction effects (multiplicative effects) using principal components. The AMMI model is expressed as follows;

(1) $$Y_{i\,j}=\mu +\delta_{i}+\beta_{j}+\sum_{k=1}^{K}\lambda_{k}\,\delta_{i\,k}\,\beta_{j\,k}+e_{i\,j}$$

where **Y_ij_** is mean of *i*^th^ genotype in *j*^th^ environment, μ is the overall mean, *δ***_i_** is the *i*^th^ genotypic effect, *β***_j_** is the *j*^th^ environmental effect, *λ***_k_** is the Eigen value for PC axis *k*, *δ***_ik_** is the principal component score ithgenotype for *k*^th^ PC axis, *β***_jk_** is the principal component score *j*^th^ environment for *k*^th^ PC axis, **e_ij_** is the error term. AMMI analysis and graphical representation through AMMI biplots was done using ‘agricolae’ r package (Mendiburu and de Mendiburu, 2019).

The equation for GGE biplots which uses site regression linear bilinear model is depicted as follows;

(2) $$Y_{i\,j}-\mu_{j}=\sum_{k=1}^{t}\lambda_{k}\,\delta_{i\,k}\,\beta_{j\,k}+e_{i\,j}$$

GGE biplots were used to determine the best environment (which-won-where pattern) for recommending specific genotypes to specific environments or seasons. GGE biplots helps to determine stable genotype(s) across the locations or seasons and to understand discriminative power among genotypes in target locations or seasons. GGE biplots were plotted using ‘GGEBiplot’ r package (Dumble, 2017).

Correlation and stepwise regression analysis was carried out in SAS Version 9.3 software available at ICAR-IIRR. The regression model in terms of matrix notation is expressed as follows;

(3) Y=X⁢β+e

Where Y is the response variable, X is the vector of exogenous variables, β is the regression coefficient vector and e is the residuals term assumed to be normally distributed with e∼N(0,σ^2).

### Genotyping by ddRAD-seq

The ddRAD-seq protocol was followed in the present study (Peterson et al., 2012). Genomic DNA was extracted using the DNeasy Plant Mini Kit (Qiagen, Hilden, Germany) from pooling five 14 days old seedlings. DNA was checked for its concentration and quality on agarose gel and Qubit fluorometer (Thermofisher Scientific, United States). Genomic DNA of 40 genotypes was double digested using restriction enzymes *Sph*I and *Mluc*I (NEB, England) and the digested products were fractionated in 2% agarose gel electrophoresis to check product size in 250–400 bp. The Agencourt AMpure XP beads clean-up technology (with Dynabeads, Invitrogen) was used to clean the digested products using standard protocols (Beckman Coulter, United States). Barcoded adapters were ligated to each DNA sample using T4 ligase (NEB, England) followed by indexing with the addition of Index-1 and Index-2 (8 nt long) for multiplexing sequencing library in NGS Illumina. To increase the concentration of sequencing libraries, PCR amplification (8–12 cycles) was performed using PhusionTM PolymeraseKit (Fisher Scientific, United Kingdom). Products of PCR amplification were analyzed in an Agilent Bio-analyzer (Agilent, United States) to quantify molarity and fragment size distribution. The size-selected library was sequenced on an Illumina HiSeq2500 (Illumina, United States). RAD tags were identified in the raw reads, processed for base trimming and removal of the Illumina adapter sequences. The high quality paired end reads were aligned to *Oryza sativa* L. cv. Nipponbare reference genome (MSU7) using.bowtie2 version 2.2.2.6 (Langmead and Salzberg, 2012). The variant calling was performed based on the aligned reads to the reference genome and identified the SNPs using SAMtools version 0.1.19 (Li et al., 2009). The SNP markers were screened based on 90% call rate, locus homozygosity, and minor allele frequency (MAF) 0.05. The variant annotation was performed based on rice gene models, using in-house pipelines VARIMAT (AgriGenome Labs Pvt., Ltd., India). The Population structure was determined by setting the number of groups (K) from 1 to 10.

### Genome Wide Association Studies

In the present study, Kinship analysis was studied according to Endelman and Jannink (2012) using Centered-IBS matrix value in TASSEL5 (Bradbury et al., 2007). Dendrogram was constructed based on similarity matrix of the SNPs variations using neighbor-joining module in TASSEL 5 program. Out of the total variants, the monomorphic SNPs were filtered out and only polymorphic SNPs in the annotated regions were considered for downstream analyses. Associations between SNPs and phenotypic data were computed using the Genome Association and Prediction Integrated Tool—GAPIT (Lipka et al., 2012) based on the Mixed Linear Model (MLM) that controls the population structure and genetic relatedness among the individuals by incorporating the Q and K matrices. Principal component analysis (PCA) was performed using GAPIT software and genome wide association between traits were calculated (Lipka et al., 2012).

In MLM, X represents the genotype and Y the phenotype (The phenotype comprised three data sets of nine traits recorded during 2014, 2015, and 2016), allowing associated values of each SNP to be calculated (Supplementary Table S2). A value of < 0.05 was used as the threshold to determine the existence of a significant association. SNPs associated with nine traits in common identified across three years evaluation were only further analyzed. The amount of phenotypic variation explained by each marker was estimated by *r*^2^. Associations were considered significant when *p* ≤ 0.01 or LOD scores greater than 4.0. The functional annotation of the associated SNPs was identified in *Oryza sativa* L. cv. Nipponbare as reference genome^4^.

### Co-localization

The positions of the associated SNPs of ZPR and SPY of the present study were compared to the genomic positions of the markers from the reported QTL to study the co-localization.

### Candidate Genes in the Region of SNPs Associated With ZPR and SPY

Based on earlier studies, the linkage disequilibrium was reported to be between 100 and 300 kbp on average for different subpopulations of rice (Zhao et al., 2011), thus a total of 600 kbp region (300 kbp each side of associated SNP) spanning each of the associated SNPs with ZPR and SPY was surveyed for the putative candidate genes^5^.

## Results

### Agro- Morphological, Yield and Mineral Traits

**FIGURE 1:**
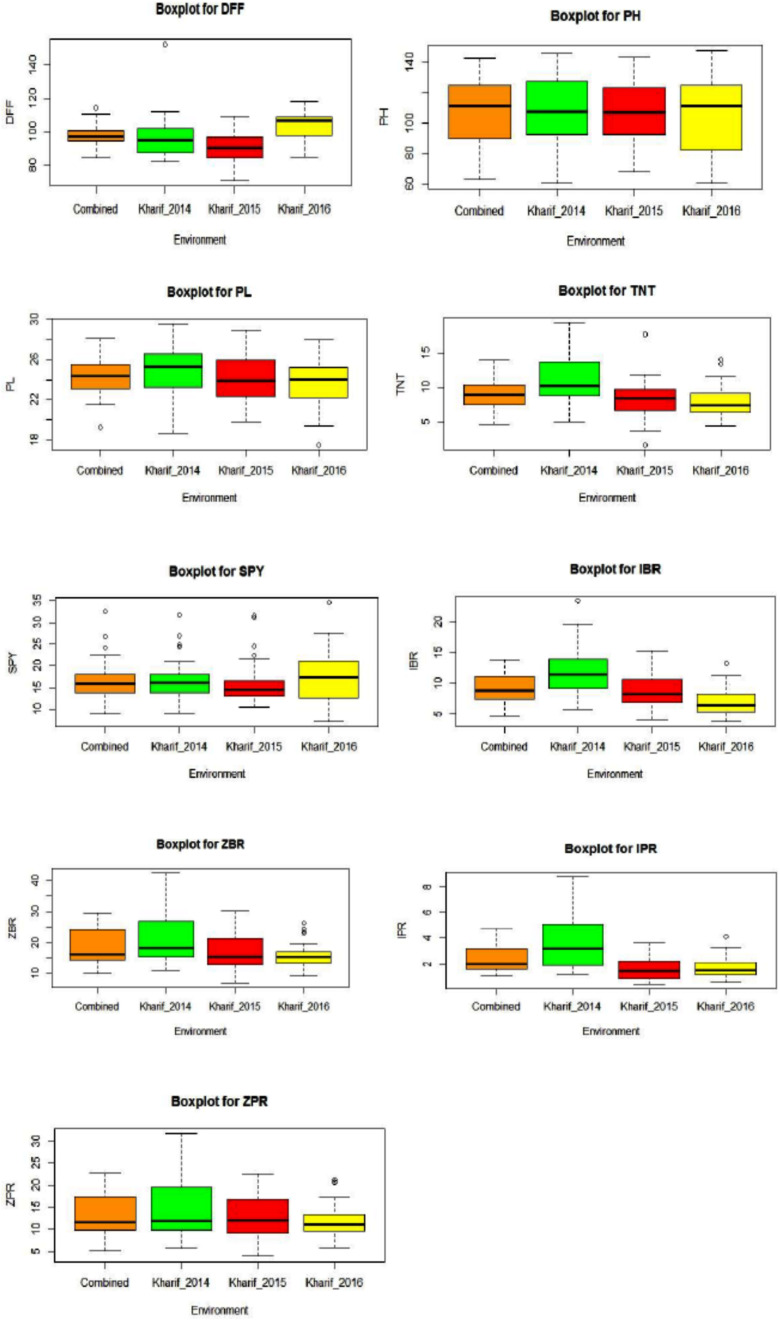
Box plots for nine traits [DFF, Days to fifty percent flowering; PH, Plant Height (cm); PL, Panicle Length (cm); TNT, Total Number of Tillers per plant; SPY, Single Plant Yield (g); IBR, Fe content in Brown Rice (ppm); ZBR, Zn content in Brown Rice (ppm); IPR, Fe content in Polished Rice (ppm); ZPR, Zn content in Polished Rice (ppm)] across three environments E1 – *Kharif* 2014, E2 - *Kharif* 2015, E3 – *Kharif* 2016 and combined across environments.

All the evaluated 40 genotypes have shown a wide range variation for the agro-morphological yield and mineral related traits within an environment and between the environments E1, E2, and E3. DFF and SPY were higher in E3 compared to E2 and E1. The trait values for PH, PL, TNT, IBR, ZBR, IPR, and ZPR were higher in E1 compared to E2 and E3. Wide range was observed for the traits mean of three environments as 84.72 to 114.17 days for DFF, 62.87 to 142.57 cm for PH, 19.24 to 28.10 cm for PL, 4.53 to 13.91 for TNT, 8.92 to 32.45 g for SPY, 4.71 to 13.78 ppm for IBR, 10.03 to 29.66 for ZBR, 1.06 to 4.74 for IPR and 5.20 to 22.65 for ZPR (Figure 1). The trait wise descriptive statistic values of three environments were presented in Supplementary Table S2. Morphological variation of seed of 40 genotypes and the frequency distribution of nine traits were presented in Supplementary Figures S1A–C. Across the three environments G26 (Kalanamak) showed maximum DFF (114 days); G37 (Sirarakhong Manui) showed highest PH (142.5cm); G34 (Jahagisim) showed longest PL (29.1 cm); G13 (Sampada) has shown maximum TNT (13); G8 (Savitri) has shown highest SPY (32.4 g). G28 (Kala Jira Jaha) has shown highest IBR (13.7 ppm); G17 (Taroari Basmati) has shown highest ZBR (29.6 ppm). G17 (Taroari Basmati) has shown highest IPR (4.7 ppm); G22 (Edavankudi Pokkali) has shown highest ZPR (22.6 ppm). The mean loss found to be 7 ppm for Fe and 5 ppm of Zn content during polishing of rice. The maximum loss was found in G32 for Fe (11.1 ppm) (Mima) and in G17 (Taroari Basmati) for Zn (10.3 ppm). Minimum loss was noted in G14 (Pushyami) for Fe (3.6 ppm) and in G9 (Pusa Basmati 1) for Zn (2.5 ppm). There were four genotypes G22 (Edavankudi Pokkali), G18 (Akut Phou), G24 (DRRDhan 45) and G21 (High Fe rice) with maximum ZPR (> 20.8 ppm). G8 (Savitri), G7 (NDR 359), G38 (Wungrei) and G13 (Sampada) were the top four genotypes for single plant yield > 26.4 g. In E1, for SPY, G8 (Savitri) showed the maximum and G26 (Kalanamak) had the minimum, whereas the highest ZPR was observed in G23 (Kadamkudi Pokkali) and the least in G14 (Pushyami). In E2, G7 (NDR 359) had maximum and G26 (Kalanamak) had minimum SPY, while G22 (Edavankudi Pokkali) has shown the highest and G14 (Pushyami) has shown the least ZPR. In E2, G8 (Savitri) had maximum and G26 (Kalanamak) minimum SPY, while G32 (Mima) has shown maximum and G14 has shown minimum ZPR (Pushyami).

### Correlations

For the mean values of nine traits across three environments, highly significant and positive correlations were found among Zn, Fe with brown and polished grains *viz*., ZBR, ZPR, IBR and IPR. For the four traits of Zn and Fe in brown and polished grains, highly significant negative correlations with SPY were observed. Similarly, DFF has also showed significant negative correlation with ZBR, IBR and IPR and also with ZPR though not significantly. Interestingly, both IPR and IBR have shown positive correlation with PH (Table 2). Environment wise correlation data was given in Supplementary Table S3.

**TABLE 2:** Pearson correlation analysis for mean values of nine traits across three environments among 40 genotypes.

	**DFF**	**PH**	**PL**	**TNT**	**SPY**	**IBR**	**ZBR**	**IPR**	**ZPR**
DFF	1								
PH	−0.246	1							
PL	0.006	0.545**	1						
TNT	−0.232	0.029	–0.125	1					
SPY	−0.086	–0.130	–0.104	–0.043	1				
IBR	−0.056	0.575**	0.263	0.013	−0.420	1			
ZBR	−0.325	0.310	0.116	0.213	−0.431	0.518**	1		
IPR	−0.321	0.391	0.231	0.277	−0.381	0.554**	0.797**	1	
ZPR	−0.293	0.288	0.134	0.093	−0.442	0.560**	0.962**	0.794**	1

**Significant *p*-Value < 0.001. DFF, Days to fifty percent flowering; PH, Plant Height (cm); PL, Panicle Length (cm); TNT, Total Number of Tillers per plant; SPY, Single Plant Yield (g); IBR, Fe content in Brown Rice (ppm); ZBR, Zn content in Brown Rice (ppm); IPR, Fe content in Polished Rice (ppm); ZPR, Zn content in Polished Rice (ppm).

### Cluster Analysis Based on Phenotype

Dendrogram of 40 genotypes based on nine traits has shown 15 clusters with cluster 1 consisting 19 genotypes, cluster 2 with four genotypes; cluster 3 with four genotypes; cluster 4 with two accessions and the remaining clustering was as single genotypes (Supplementary Figure S2).

### Stability of Genotypes Across the Environments

Combined ANOVA for the data of 40 genotypes across three environments indicated significant variance and mean sum of squares for genotype as well as for genotype × environment effect (G × E) for nine traits of the study (Supplementary Table S4). As important target traits for the development of biofortified rice varieties, the results were elaborated only for ZPR and SPY.

### ZPR

**FIGURE 2:**
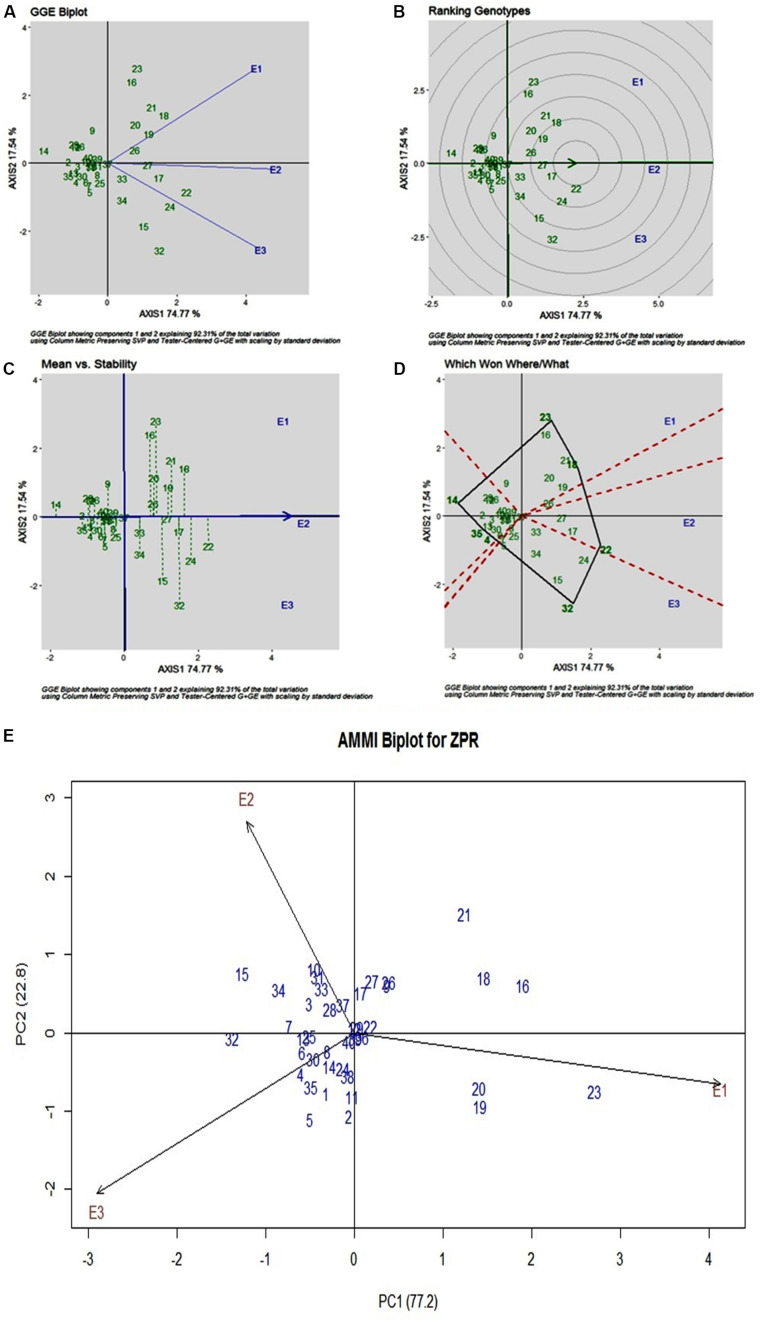
**(A)** GGE Biplot for Zn content in polished rice (ZPR). **(B)** GGE Biplot for Zn content in polished rice (ZPR)-Ranking of Genotypes. **(C)** GGE Biplot for Zn content in polished rice (ZPR) – Mean v/s Stability. **(D)** Which Won Where/What plot for Zn content in polished rice (ZPR). **(E)** AMMI Biplot for Zn content in polished rice (ZPR).

Stability analysis of ZPR across all the environments has shown genotypic (G) effect was 66.66%, environment (E) effect was 3.93%, and genotype and environment (G × E) effect was 27.43% (Table 3). Three environments showed almost equal discrimination power, whereas E2 was found as the representative environment (Figure 2A); as it falls near to the Average-Environment Axis. The G37 (Sirarakhong Manui) was found near the origin was the less interactive genotype. G23 (Kadamakudi Pokkali) and G18 (Akutphou) were found to be the best in E1, whereas G22 (Edavankudi Pokkali) was found to be the best in E2 and E3, and G32 (Mima) was found to be the best in E3 (Figure 2B). Based on the mean vs stability G22 (Edavankudi Pokkali), G17 (Taraori Basmati), G27 (Chittimuthyalu) and G26 (Kalanamak) were found to be stable among the given three environments, whereas G32 (Mima) and G23 (Kadamakudi Pokkali) found more unstable genotypes (Figure 2C). The graph Which Won Where/What (Figure 2D) clearly explains that the G18 (Akut Phou) won in E1, G22 (Edavankudi Pokkali) won in E2 and E3, G32 (Mima) won in E3. The AMMI biplot shows PC1 contributes 77.2% variability and PC2 contributes 22.8% variability (Figure 2E).

**TABLE 3:** Analysis of variance of Zn content in Polished Rice (ZPR); Single Plant Yield (SPY) of 40 genotypes across three environments.

**Source**	**DF**	**SS**	**MSS**	**F Value**	**Pr > F**	**R-Square**	**SS(%)**
**ZPR**							
Model	125	10262.72	82.10	443.11	<0.0001	0.995	99.58
Env	2	405.45	202.72	1094.12	<0.0001		3.93
Rep(Env)	6	160.76	26.79	144.60	<0.0001		1.56
Genotypes	39	6870.07	176.16	950.73	<0.0001		66.66
Env*Genotypes	78	2826.44	36.24	195.57	<0.0001		27.43
Error	234	43.36	0.19				0.42
Corrected Total	359	10306.07					100.00
**SPY**							
Model	125	9486.06	75.89	88.88	<0.0001	0.979	97.94
Env	2	125.73	62.87	73.63	<0.0001		1.30
Rep(Env)	6	180.70	30.12	35.27	<0.0001		1.87
Genotypes	39	7142.86	183.15	214.51	<0.0001		73.75
Env*Genotypes	78	2036.78	26.11	30.58	<0.0001		21.03
Error	234	199.79	0.85				2.06
Corrected Total	359	9685.85					100.00

### SPY

**FIGURE 3:**
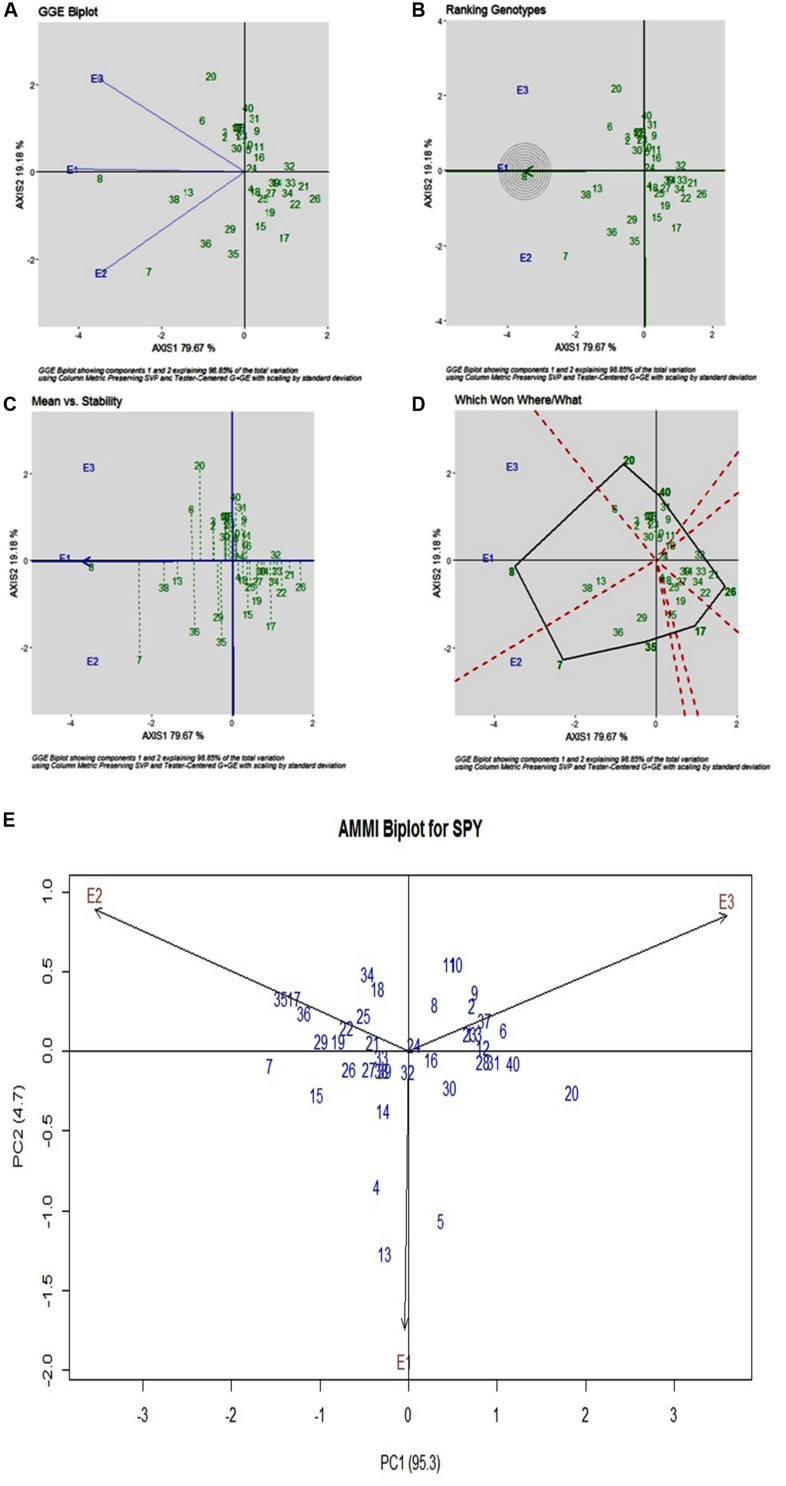
**(A)** GGE Biplot for Single Plant Yield (SPY). **(B)** GGE Biplot for Single Plant Yield (SPY)-Ranking of Genotypes. **(C)** GGE Biplot for Single Plant Yield (SPY) – Mean v/s Stability. **(D)** Which Won Where/What plot for Single Plant Yield (SPY). **(E)** AMMI Biplot for Single Plant Yield (SPY).

For SPY, across three environments, genotypic (G) effect was 73.75%, environment (E) effect was 1.30%, and genotype and environment (G × E) effect was 21.03% (Table 3). All three environments showed almost equal discrimination power, whereas E1 was found as the representative environment (Figure 3A), as it falls on the Average-Environment Axis. G24 (DRRDhan 45) was found near the origin and was the less interactive genotype. The G8 (Savitri) was found to be the best in E1, whereas G7 (NDR359) was found to be the best in E2 (Figure 3B). Based on the mean *vs* stability G8 (Savitri) was found more stable among three environments, whereas G7 (NDR359) and G20 (Tilakkachari) found more unstable genotypes (Figure 3C). The graph Which Won Where/What clearly explains that the G8 (Savitri) won in E1 and E3, whereas G7 (NDR359) won in E2 (Figure 3D). The AMMI biplot shows PC1 contributes 95.3% variability and PC2 contributes 4.7% variability (Figure 3E).

### Stepwise Regression Analysis of ZPR and SPY

Stepwise regression analysis for grain Zn polished rice (ZPR) was carried out to identify the factors influencing ZPR and regression equation for ZPR is depicted as followed.

(4) $$\hat{\mathrm{ZPR}}=1.068+0.73\,\mathrm{ZBR}-0.27\,\mathrm{TNT}+0.510\,\mathrm{IPR}$$

Factors like ZBR (92.56%), TNT (1.31%) and IPR (0.4%) explain 94.3% variation in the model (Table 4a). For every one-ppm increase in ZBR, there is 0.73 ppm increase in ZPR, thus ZBR has an expected positive effect on ZPR. For every one-tiller increase in TNT, there was a 0.27 ppm decrease in ZPR, thus TNT has negative effect on ZPR. For every one ppm increase in IPR, there was 0.51 ppm increase in ZPR, thus IPR has positive effect on ZPR.

**TABLE 4a:** Stepwise Regression Analysis of ZPR.

**Variable**	**Parameter Estimate**	**Standard Error**	**Probability**	**Partial R-Square**	**Model R-Square**
Intercept	1.068	0.929	0.2577		0.943
ZBR	0.732	0.053	< 0.0001	0.9256	
TNT	–0.278	0.088	0.0033	0.0131	
IPR	0.510	0.299	0.0974	0.0046	

For SPY, the resultant regression equation is as followed

(5) $$\hat{\mathrm{SPY}}=40.83-0.18\,\mathrm{DFF}-0.52\,\mathrm{ZPR}$$

Factors *viz*., DFF (19.52%) and ZPR (5.7%) explained 25% variation in the model and rest of the variations may be due to other factors which were not considered in this study (Table 4b). In terms of individual factors, for every one ppm increase in ZPR, there was a −0.52 g decrease in SPY, implying negative impact of ZPR on SPY. And also for every one day increase in DFF, there was −0.18 g decrease in SPY, indicating that the DFF has negative effect on SPY.

**TABLE 4b:** Stepwise Regression Analysis of SPY.

**Variable**	**Parameter Estimate**	**Standard Error**	**Probability**	**Partial R-Square**	**Model R-Square**
Intercept	40.83	11.76	0.0013		0.25
DFF	–0.18	0.11	0.1234	0.1952	
ZPR	–0.52	0.15	0.0015	0.0507	

*DFF, Days to fifty percent flowering; TNT, Total Number of Tillers per plant; SPY, Single Plant Yield(g); ZBR, Zn content in Brown Rice (ppm); IPR, Fe content in Polished Rice (ppm); ZPR, Zn content in Polished Rice (ppm).*

### Genotyping by Sequencing ddRAD

Out of 65,670,220 total reads, 52,162,603 reads (90.4%) were aligned with 47,221,247 unique reads (94.7%) (Supplementary Table S5). A total of 481,984 variants were called after filtering out duplicated reads with a mean sequence length of 100 bp and phred quality score ≥ 30. Only 452,550 uniquely mapped reads were used for identification of the SNPs. Raw data generated for this study was submitted to the sequence read archive at NCBI under BioProject No: PRJNA626560.

### Genome-Wide Discovery of SNPs

Considering variants only at read depth (RD) 10 chromosome wise of 40 genotypes, the range was from 20,531 (chromosome 9) to 43,143 (chromosome 1) for SNPs (Supplementary Figure S3) and SNP variants were not uniformly distributed among 12 chromosomes. The variant density was estimated to be 95 SNPs per 100 Kb in comparison with MSU release 7 assembly. Chromosome 2 shows the highest SNP density (111/100 Kb), while the lowest SNP density (81/100 Kb) was found in chromosome 4 (Supplementary Table S6). Out of the total 390,346 SNP variants identified, ranged from 7 (G15 Jalpriya) to 34,670 (G10 Jaya) **(**Supplementary Table S6). On pair wise comparisons of polymorphic SNPs, lowest (540) between G24 (DRRDhan 45) and G25 (HP5) and highest between G24 (DRRDhan 45) and G32 (Mima) were observed **(**Supplementary Table S7). A majority of the SNP variants (95.7%) were found to be homozygous. Most of the SNP changes transitions (A/G and C/T) than transversions with Ts/Tv ratio of 1.52 (Supplementary Table S8).

### Annotation of SNP Variants in the Genomic Regions

**FIGURE 4:**
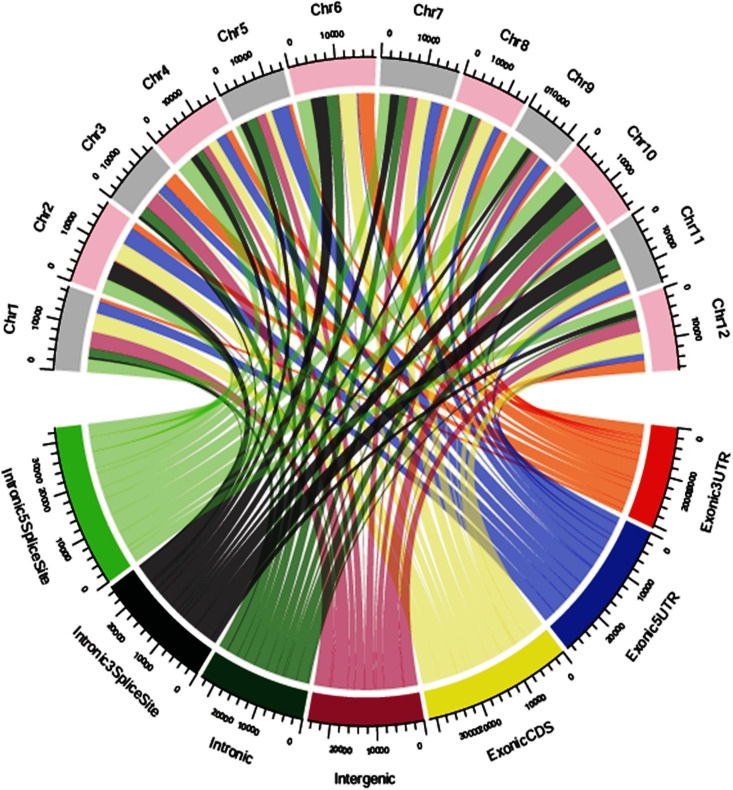
Genomic position of distribution of SNPs chromosome wise.

The SNP variants were annotated using an in-house pipeline against the gene model provided by MSU release 7. A total of 39,137 polymorphic SNPs variants in the annotated regions were identified across 40 genotypes. Among the total SNPs, 65.95% were intergenic, 16.72% were intronic, 12.95% were in exonic-CDS, 2.82% were in exonic-3UTR, 0.78% were in exonic-5UTR, 0.43% were in intronic-5 splice site and 0.35% in intronic-3 splice site regions (Supplementary Figure S4). Across 12 chromosomes, the polymorphic SNPs ranged from 2,320 to 4,388 with a mean of 3,261. The identified SNPs in the exonic-3UTR region were 1,104 and ranged from 34 to 150 with a mean of 92 SNPs per chromosome. In the exonic-5 UTR region, a total of 304 SNPs were identified ranging from 5 to 48 with a mean of 25 SNPs per chromosome. A total of 5,070 SNP variants were identified in the exonic-CDS regions with a range from 309 to 558 with a mean of 423 SNPs per chromosome. A total of 6,544 SNP variants were distributed in intronic regions ranging from 366 to 784 with a mean of 545 SNPs per chromosome. In intronic-3 splice site regions, 138 SNPs were reported and a minimum of 5 SNPs and maximum of 18 SNPs per chromosome were distributed with a mean of 12 SNPs. There were 168 SNPs identified in intronic-5 splice site regions with a mean of 14 SNPs per chromosome which ranged from 19 to 14 SNPs per chromosome. In the intergenic regions, a total of 25,809 SNP variants were identified with minimum of 1,518 SNPs and maximum of 2,902 SNPs per chromosome and with a mean of 2,151 SNPs (Figure 4 and Supplementary Table S9).

### Cluster Analyses Based on Genotyping

Based on 39,137 SNPs, 40 genotypes were separated into three distinct groups, with 19 genotypes as one group, and the other two groups with 16 and five genotypes. Two diverse groups were identified by principal component analysis with well separated lines (Supplementary Figure S5). The clustering appears to be coincided with PH *viz*., group 1 with 93.8 cm); group 2 (124.9 cm) and group 3 (102.8 cm). Modest concurrence of clustering was also observed for ZPR as mean for group1 to be 10.8 ppm, group 2 to be 15 ppm and group 3 to be 15.8 ppm (Supplementary Table S10). It was observed that out of 19 genotypes in group 1, 12 genotypes were high yielding varieties.

### Association of SNPs With Phenotype

Genome Wide Association Study using 39,137 SNPs from 40 genotypes with phenotype data of three seasons showed that multiple regions are associated with the traits under study ZPR, ZBR, IPR, IBR, SPY, PL, NT, PH, and DFF. Considering the common SNPs across the three years, a total of 188 SNPs (P < 0.01) were found and were classified based on their location in exonic, exonic UTR, intronic splice sites, intronic and intergenic regions (Table 5). Maximum number of associated SNPs was distributed by intergenic region with 63.8%, followed by intronic region with 18.6%, exonic CDS region with 15.4% and a least of 1.1% SNP in exonic UTR region and intronic-5splice_site region. The associated SNPs with traits and positions are detailed in Supplementary Table S11. The chromosome 7 has shown highest number of SNPs (50) and lowest SNPs (2) were identified on chromosomes 5 and 8 with a mean of 15.7 SNPs per chromosome.

**TABLE 5:** Stable SNPs associated with six traits common across the three seasons.

**Trait**	**Exonic CDS**	**Exonic UTR**	**Intronic-5** **splice_Site**	**Intronic**	**Intergenic**	**Total**
ZPR	4			1	5	10
ZBR	7		1		13	21
IPR	–	–	–	–	1	1
IBR	–	–	–	–	–	0
SPY	6	–	–	11	20	37
PL	4	1	1	11	6	23
NT	–	–	–	–	–	0
PH	8	1	–	12	75	96
DFF	–	–	–	–	–	0
**Total SNPs**	29	2	2	35	120	188

*DFF, Days to 50% flowering; TNT, Total Number of Tillers per plant; SPY, Single Plant Yield(g); ZBR, Zn content in Brown Rice (ppm); IPR, Fe content in Polished Rice (ppm); ZPR, Zn content in Polished Rice (ppm); CDS, coding sequences; UTR, untranslated region.*

### Association of SNPs in Putative Candidate Genes With SPY and ZPR

**FIGURE 5:**
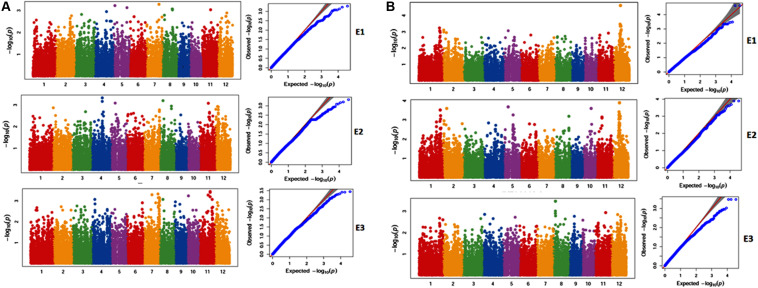
**(A)** Selected Manhattan and QQ plots of associated SNPs across 40 genotypes for Zn content in polished rice (ZPR). **(B)** Selected Manhattan and QQ plots of associated SNPs across 40 genotypes for Single Plant Yield (SPY).

A total of 188 SNPs were found to be associated with six traits across three environments and their genomic positions were detailed in Supplementary Table S11. Only the candidate genes associated ZPR and SPY are discussed in detail. The Manhattan and QQ plots of associated SNPs across 40 genotypes for ZPR and SPY were presented in Figures 5A,B. Out of three candidate genes found to be associated for ZPR, SNP (A > G) was located in intronic region of LOC_Os03g47980 encoding armadillo like protein and it explained 17% of phenotypic variance (PV). Another SNP (T > C) was located in the exonic CD of LOC_Os07g48050 encoding peroxidase and found to be silent mutation (TGT > TGC) explaining 19% of PV. The third candidate gene LOC_Os07g47950 encoding protein found to be most interesting explaining 23% of PV with three SNPs *viz*., G > A (GCG > ACG), a missense mutation changing alanine to threonine; G > A (GCG > GCA), a silent mutation and C > T (CTC > TTC), a missense mutation changing leucine into phenylalanine (Table 6).

**TABLE 6:** Significant SNPs located in candidate genes associated with ZPR and SPY along with phenotypic variance (PV%).

**S No**	**Gene ID**	**Chromosome**	**Marker ID**	**Genic region**	***R*-square**	**PV %**
**ZPR**						
1	LOC_Os03g47980 (-)	3	C3.27280585	Intronic	0.178590206	17
2	LOC_Os07g47950 (+)	7	C7.28641194	Exonic-CDS	0.24118621	23
		7	C7.28641196	Exonic-CDS	0.24118621	
		7	C7.28641209	Exonic-CDS	0.217452607	
3	LOC_Os07g48050 (+)	7	C7.28688976	Exonic-CDS	0.197142974	19
**SPY**						
1	LOC_Os01g64960 (+)	1	C1.37698648	Intronic	0.206905872	21
2	LOC_Os01g64890 (+)	1	C1.37665805	Exonic-CDS	0.166038992	17
3	LOC_Os06g30070 (+)	6	C6.17331199	Exonic-CDS	0.183732247	18
4	LOC_Os12g19090 (-)	12	C12.11083190	Intronic	0.254497827	25
5	LOC_Os12g19350 (-)	12	C12.11224184	Intronic	0.247276479	25
6	LOC_Os12g19590 (+)	12	C12.11423711	Intronic	0.186273816	19
7	LOC_Os12g21500 (+)	12	C12.12083507	Intronic	0.247276479	25
8	LOC_Os12g22300 (-)	12	C12.12589709	Intronic	0.247276479	25
9	LOC_Os12g19370 (+)	12	C12.11245053	Intronic	0.247276479	22
		12	C12.11245866	Exonic-CDS	0.185023395	
10	LOC_Os12g19890 (-)	12	C12.11588978	Exonic-CDS	0.362240391	36
11	LOC_Os12g20420 (-)	12	C12.11924838	Exonic-CDS	0.244926128	24

A total of 11 genes has shown association with SPY, from which 6 genes has shown the intronic mutations *viz.* (1) LOC_Os01g64960 -chlorophyll A-B binding protein, putative, expressed gene has intronic mutation of C/A. (2) LOC_Os12g19090-metalloprotease ATP23, putative, expressed has intronic variation C/T. (3) LOC_Os12g19350-expressed protein has intronic mutation of A/G, (4) LOC_Os12g19590 - WD domain, G-beta repeat domain containing protein, has shown intronic mutation of A/T, (5) LOC_Os12g21500- exosome complex exonuclease, putative, expressed has change in intronic region with G/T, and (6) LOC_Os12g22300 - retrotransposon protein, putative, Ty3-gypsy subclass, expressed gene has intronic SNP A/G. Another gene LOC_Os12g19370 encoding DNA polymerase I family protein, has an intronic mutation with A/G and exonic-CD silent mutation of GGC > GGA. A gene LOC_Os01g64890 coding for CorA-like magnesium transporter protein, putative has a silent mutation TCT > TCA in exonic-CD. Three genes have shown missense mutation in exonic-CD region. LOC_Os06g30070 encoding retrotransposon protein, putative, Ty3-gypsy subclass gene has expressed a missense mutation (CCT > CTT) in exonic-CDS region, thus amino acid proline was replaced by leucine. The second gene LOC_Os12g19890 also encodes retrotransposon protein, putative, LINE subclass and has an Exonic-CD region missense mutation (GAG > AAG) changing glutamic acid by lysine. The third gene LOC_Os12g20420 also encoding transposon protein, putative, unclassified, ha also shown a missensed mutation (CGT > TGT) at exonic-CD region changing arginine to cysteine (Table 6).

### Co-localization of QTL

Out of three associated SNPs located in candidate genes for ZPR, two genes on chromosome 7 were co-localized with earlier reported genomic region with rMQTL7.1 (Raza et al., 2019). The associated candidate gene on chromosome 3 found to be novel. Interestingly, none of the SNPs associated with SPY were found to be co-localized with earlier reported genomic regions.

### Genes in the Proximity Regions of (∼600 kbp) Associated SNPs With ZPR and SPY

For ZPR, for the associated SNPs of the candidate genes (LOC_Os07g47950 and LOC_Os07g48050) on chromosome 7, ∼140 genes were noted and for LOC_Os03g47980, 90 genes were found. For SPY, 143 genes on chromosome 1, 82 genes on chromosome 6 and 328 genes on chromosome 12 were observed (Supplementary Table S12).

## Discussion

Identification of environmentally stable donor lines for high grain Zn would contribute and accelerate the development of biofortified Zn rice varieties. Several landraces have been identified with grain Zn up to 58.4 ppm in brown rice (Gregorio et al., 2000; Norton et al., 2014; Tan et al., 2020) and up to 40.9 ppm in polished rice across world since 2000 (Lee et al., 2008; Bollinedi et al., 2020; Rao et al., 2020). But reports on validation of the identified landraces with high grain Zn for their stability across environments and systematic deployment of identified landraces as donors in breeding of Zn biofortified rice varieties are limited. Stable donor/s identified for the trait of interest can hasten the development of breeding lines/varieties in rice as demonstrated for salinity tolerance from donor ‘Pokkali,’ submergence tolerance from donor ‘FR13A,’ phosphorus uptake from ‘Kasalath,’ aroma and grain quality from ‘Basmati’ donors (Septiningsih et al., 2009; Chin et al., 2011; Singh et al., 2018; Rana et al., 2019). In the present study, we identified four stable promising donors (Edavankudy Pokkali, Taraori Basmati, Chittimuthyalu and Kalanamak) for high grain Zn > 28 ppm in polished rice for the development of Zn biofortified varieties. Aromatic genotypes like Basmati were reported to be high in grain Zn in earlier studies of International Rice Research Institute (IRRI) (Gregorio et al., 2000). G27 Chittimuthyalu and G26 Kalanamak are included as check genotypes for high grain Zn in the Biofortification trials of rice varietal release program in India through All India Coordinated Rice Improvement Project (AICRIP)^6^ (Rao et al., 2020).

In the present study, for released widely adopted popular varieties, the mean value of grain Zn in polished rice was found to be only 14 ppm ranging from 10.2 ppm (G14 Pushyami) to 16.5 ppm (G9 Pusa Basmati 1 and G8 Savitri). Most of the released rice varieties are known to be less in grain Zn content ranging from < 12 to 14 ppm in polished rice (Swamy et al., 2016; Mahender et al., 2016). Akut Phou (G18), a variety included in the present study appears to be already biofortified with polished grain Zn > 24 ppm and reported yield of 4–6 tons/ha. The variety was released from Manipur state of India during 1990 and is only locally popular for its grain quality. Moderate levels of higher grain Zn was also observed for widely adopted rice varieties like Savitri (G8) and Pusa Basmati 1 (G9). Thorough evaluation of released rice varieties for the grain Zn could lead to the identification of base material with proven yield and a possible higher Zn content.

Wide genetic variability was observed for the nine traits of the study considering the diversity of the genetic material comprising released varieties and breeding lines including developed for high grain Zn and landraces from various geographical regions of India (Table 1). For IPR, maximum value of 8 ppm was obtained in polished rice, whereas the recommended content is 12 ppm by HarvestPlus^1^. Correlation analyses of the mean values of three environments in the present study corroborated the reported significant positive correlations among Zn and Fe of brown and polished grains and significant negative correlations of grain Zn and Fe with yield (Gao et al., 2006; Norton et al., 2010; Anuradha et al., 2012; Gangashetty et al., 2013; Sathisha, 2013; Inabangan-Asilo et al., 2019). Significant negative correlation of DFF and positive correlation of PH with grain Zn and Fe observed in the study need to be confirmed with large set of genotypes. While there was no significant correlation observed between PL with grain Zn and Fe in our study, Descalsota et al. (2018) reported PL had direct effect on grain Fe and Zn. Significant positive correlation between grain Fe and Zn with plant height and positive correlation between grain Fe and Zn with days to maturity was reported by the same group was also earlier reported by Inabangan-Asilo et al. (2019). The wide range in duration of the genotypes of study, especially of landraces would have contributed toward negative association of DFF with grain Fe and Zn. Stepwise regression analyses clearly depicted the expected positive contribution of ZBR and IPR and negative influence of total number of tillers (*R*^2^ = 0.943) for enhanced ZPR and only ZBR on shown very close association (*R*^2^ = 0.92) (Tables 4a,b). The loss of grain Zn during polishing is due to the loss of aleurone layer, which was reported to be significant for mineral content in rice (Sellappan et al., 2009). Previous studies reported a loss to the tune of 5 to 30% of grain Zn during the polishing in germplasm and mapping populations (Lee et al., 2008; Rao et al., 2020). Two significant conclusions from stepwise regression analysis in the present study were the determination of threshold of grain Zn in brown rice *viz*., for fulfilling international threshold value of rice grain Zn content (28 ppm), the developers may need to target ≥ 38.5 ppm increase in ZBR. Based on germplasm screening for grain Zn in brown and polished rice, our group suggested germplasm with zinc content ≥ 35 mg/kg in brown rice can be promising based on the threshold value of 28 mg/kg by HarvestPlus and 19.0% overall mean loss of zinc during polishing (Rao et al., 2020). Thus, the value of 38.5 ppm of Zn in brown rice obtained through regression analysis in the present study also confirms our earlier study. And the second observation was negative association (*R*^2^ = 0.25) with DFF (*R*^2^ = 0.19) and ZPR (*R*^2^ = 0.5) of SPY suggesting ZPR has only limited negative effect on grain yield and the possibility of simultaneous improvement of yield and grain Zn content as suggested by earlier studies (Swamy et al., 2016; Pradhan et al., 2020). The impact of soil factors on rice grain Zn content was earlier demonstrated through stepwise regression analysis (Pandian et al., 2011).

The influence of plant, soil and climate factors compounds the development of biofortified Zn rice varieties. Many soil criteria like pH, composition, mineral content, biome and agronomic practices like fertilization and irrigation are known to impact the Zn uptake and metabolism in rice plant (Gregorio et al., 2000; Wissuwa et al., 2008; Chandel et al., 2010; Rerkasem et al., 2015). Varying genotypic grain Zn content across locations was widely reported in rice underscoring the importance of identifying stable donors for grain Zn (Norton et al., 2014; Rao et al., 2020). In the present study, based on the stability and G x E interaction analyses of 20 shortlisted genotypes across three years (E1-E3), five promising donors were identified for grain Zn (ZPR) *viz*., G18 (Akut Phou), G22 (Edavankudi Pokkali), G17 (Taraori Basmati), G27 (Chittimuthyalu) and G26 (Kalanamak). For SPY, G8 (Savitri) was identified as stable genotype across environments. AMMI and GGE biplot models suggested the stable performers across the environments and partitioned the total phenotypic variance into individual factors (Gauch, 2006). Both ZPR and SPY showed maximum contribution from genotype because of the genetic material included in the present study. Through Which Won Where/What plot, common winner could not be found for ZPR, whereas G8 (Savitri) won in two environments for yield. Stability and G x E analysis of eight Zn-biofortified rice breeding lines evaluated in four seasons and eight to nine locations also identified different ideal genotypes for yield and Zn in brown rice (Inabangan-Asilo et al., 2019). Significant G × E interactions were observed among a set of 37 diverse genotypes evaluated in three environments during wet season (kharif), four stable genotypes were identified each for grain Fe and Zn based on regression (Ajmera et al., 2017). Significant G × E interactions were also reported for grain Fe in a set of 10 genotypes screened at eight environments using the AMMI-biplot and a single stable genotype was identified (Suwarto, and Nasrullah, 2011). Analysis of G x E interaction experiment conducted at same location (ICAR-IIRR) for a set of 14 backcross introgression lines (BILs) for yield related traits during two wet seasons (*Kharif)* and one dry season (*Rabi*) using AMMI, GGE biplot and *Ysi* stastics identified two stable lines with high yield (Balakrishnan et al., 2016). Based on the mean *vs* stability, three landrace genotypes *viz*., G22 (Edavankudi Pokkali), G17 (Taraori Basmati), G27 (Chittimuthyalu) and G26 (Kalanamak) were found to be stable among the given three environments suggesting their utility as donors for high grain Zn. The stable genotype for SPY G8 (Savitri) is one of the mega varieties known for its high yield in several parts of India (Robin et al., 2019). G8 (Savitri) performed well in E1 and E3 when compared to E2 indicating the influence of environmental factors such as temperature, solar radiation and sunshine, relative humidity and wind velocity. G22 (Edavankudi Pokkali) was found stable for E2 and E3 based on GGE biplot and Which Won Where What (W4). Moreover W4 explains G18 (Akut Phou) was stable in E1. The G27 (Chittimuthyalu), G26 (Kalanamak) and G17 (Taraori Basmati) were closer to Mean vs Stability zero line and based on ranking genotypes these genotypes were shown to be relatively stable genotypes. G22, G18, G27, G26 and G17 were stable for ZPR and can be used as donors in breeding programs for developing high Zn genotypes. For SPY, G8 was stable genotype based on GGE biplot, closer to Mean vs Stability zero line and W4 explains G8 was stable in E1 and common stable genotype in E2 and E3. Thus G8 can be used in future plant breeding programs as a donor for SPY.

With the advent of NGS technologies, development of genome wide SNPs in the target genotypes despite the number has become feasible. ddRAD sequencing facilitates the identification of SNPs by reducing genomic complexity and has been successfully demonstrated in rice and other crops (Yang et al., 2016; Sudan et al., 2019). In the present study, a pair of restriction enzymes digestion *(Sph*I *and MlucI)* was used for the discovery of unique SNPs following the standard procedures *viz*., raw reads de-multiplexing, sequence quality analysis, SNP calling and genotyping (Peterson et al., 2012). A total of 39,137 high quality polymorphic SNPs variants were identified in 40 rice genotypes and annotated with rice genome to identify the region, function and significance. Across chromosomes, ∼1 SNP/1 Kb was observed in the present study, with maximum number of SNPs in intergenic (65.95%), followed by intronic (16.7%), exonic or CDs (12.9%) and intronic-3 splice site (0.35%) regions. Similar trend of SNP localization in the rice genome was earlier reported (Feltus et al., 2004; Ali et al., 2018). The grouping of genotypes based on SNPs showed moderate consensus with the phenotype values of clusters suggesting the contribution of genomic regions/SNPs to phenotypic response as observed in rice introgression lines with tolerance to multiple biotic and abiotic stresses (Ali et al., 2018). Interestingly, five genotypes *viz*., G7 (Narendradhan 359), G8 (Savitri), G16 (ARB-45), G18 (Akut Phou) and G23 (Kadamakudy Pokkali) in cluster 3 found to be promising for DFF, NT, SPY, IBR, ZBR and ZPR, thus they could be deployed in the future breeding program (Supplementary Table S10). The information about pairwise combinations between promising donors and popular varieties can be further deployed for mapping the target traits (Supplementary Table S7). Association analysis of polymorphic SNPs has shown 188 stable SNPs (p < 0.01) significantly associated with six traits *viz*. IPR (1), ZPR (10), ZBR (21), PL (23), SPY (37) and PH (96) across the three environments. Considering only the common SNPs across three environments increased the reliability of identified SNPs and compensated lesser number genotypes of study. While there were a total of 1952 associated SNPs for ZPR across three years (489 in E1; 539 in E2 and 564 in E3), only stable 10 SNPs were selected based on their stability. Similarly, only 37 common stable SNPs were studied from a total of 1319 associated SNPs found (436 in E1; 44 in E2 and 395 in E3 for SPY). Similar studies of 17 marker-phenotype associations were reported for grain Fe and Zn in 102 rice genotypes with 25 gene specific and 75 SSRs (Pradhan et al., 2020). Genotyping of 175 accessions with 155 SSRs revealed 60 marker- trait associations with eight grain elements (Nawaz et al., 2015). Association mapping of 378 accessions with 143 gene specific and SSR markers identified 20 QTL for five grain elements (Huang et al., 2015). GBS analyses of 144 rice MAGIC Plus lines using 14,242 SNP markers identified 57 significant genomic regions associated with various traits including nutritional quality (Descalsota et al., 2018). Several subspecies significant specific association loci for grain mineral concentration were reported through GWAS of *indica* and *japonica* accessions (Zhang et al., 2018; Tan et al., 2020). GWAS of ∼300 accessions with 36,901 SNPs identifies 16 associations for three grain elements as top 1% most significant in at least four of the five field sites (Norton et al., 2014).

Out of 10 stably associated SNPs for ZPR, three markers were located in exonic-CDS region encoding three putative candidate genes C3.27280585 (LOC_Os03g47980), C7.28641194 (LOC_Os07g47950) and C7.28688976 (LOC_Os07g48050). While the association on chromosome 3 appears to be novel, the identified SNPs around ∼28 Mb on chromosome 7 have shown consistent co-localization with genomic regions grain Zn in rice reported through association and biparental mapping (Lu et al., 2008; Huang et al., 2015; Zhang et al., 2018). Recently, a meta QTL rMQTL7.1 has also been detected in this region through meta-analysis (Raza et al., 2019). The identification of consistent co-localized genomic region on chromosome 7 asserts the marker-trait associations in the present study. The identified genomic regions explaining moderate PV ranging from 17 – 23% can be pyramided during the breeding of biofortified Zn rice varieties. Candidate gene analyses in the flanking region (∼300 Kb upstream and downstream) of the mapped SNPs showed several genes and gene families involved in the uptake, transport, and accumulation of Zn in plants along with several hypothetical proteins. The results clearly demonstrate the utility of GBS for identification of significantly associated variant loci might involve in the Zn accumulation mechanism of rice. Twelve stable SNP markers associated with SPY were detected on chromosome 1, 6, and 12 explaining 16 to 36% of PV (Supplementary Table S12). Interestingly, co-localized genomic regions could not found out for these 12 associated SNPs for yield suggesting the possibility of their novelty. Pairwise polymorphic SNPs identified between the donors and high yielding varieties in the study are being utilized for mapping of grain Zn in the recombinant inbred lines developed between donors and high yielding varieties (Rao et al., 2020).

## Conclusion

Deploying AMMI model and GGE biplots for analyses of 20 landraces and 20 popular rice varieties across three environments, four stable landraces (Edavankudi Pokkali, Taraori Basmati, Chittimuthyalu, and Kalanamak) were identified as donors for high grain Zn for rice biofortification breeding program. Using regression analysis, contributing factors for ZPR were identified and threshold levels for ZBR for 28 ppm of ZPR were estimated. Through GBS-dd-RAD analyses, 188 stable SNPs with their locations in the genes across three environments were identified for six traits. Functionality of identified SNPs in the three candidate genes identified for ZPR and 11 candidate genes identified for SPY has been analyzed through their location in the gene. The genomic region for ZPR on chromosome 7 has co-localized with reported metaQTL region (rMQTL_7_._1_) and is a promising region for marker assisted introgression.

## Data Availability Statement

The data has been submitted to NCBI and the accession number has been provided in the manuscript as submitted to the sequence read archive at NCBI under BioProject No: PRJNA626560.

## Author Contributions

CN conceptualized the idea. PB, KS, and UC conducted the field experiments. CN, PB, SR, GU, NC, and VL carried out the data analysis. PB and CN prepared the manuscript. CN, LR, and SV edited the manuscript. All authors contributed to the article and approved the submitted version.

## Conflict of Interest

VL was employed by company AgriGenome Labs Pvt. Ltd. The remaining authors declare that the research was conducted in the absence of any commercial or financial relationships that could be construed as a potential conflict of interest.

## Footnotes

1 www.harvestplus.org

2 www.icar.iirr.org

3 www.rkmp.co.in

4 http://plantbiology.msu.edu/pub/data/Eukaryotic_Projects/o_sativa/annotation_dbs/pseudomolecules/version_7.0/all.dir

5 http://rice.plantbiology.msu.edu

6 http://www.icar-iirr.org/

## Supplementary Material

FIGURE S1A Morphological variation of seed of 40 genotypes in the study.

FIGURE S1B Frequency distribution plots for DFF-Days to 50% flowering; PH- Plant Height(cm); PL- Panicle Length(cm); NT- Number of Tillers per plant and SPY- Single Plant Yield (g).

FIGURE S1C Frequency distribution plots for IBR-Iron content in Brown Rice (ppm); ZBR- Zinc content in Brown Rice (ppm); IPR- Iron content in Polished Rice (ppm); ZPR- Zinc content in Polished Rice (ppm).

FIGURE S2 Cluster dendrogram of 40 genotypes based on nine traits (DFF-Days to fifty percent flowering; PH- Plant Height (cm); PL- Panicle Length (cm); TNT- Total Number of Tillers per plant; SPY- Single Plant Yield (g); IBR-Fe content in Brown Rice (ppm); ZBR- Zn content in Brown Rice (ppm); IPR- Fe content in Polished Rice (ppm); ZPR- Zn content in Polished Rice (ppm).

FIGURE S3 Chromosome wise total SNPs of 40 genotypes at Read Depth 10.

FIGURE S4 Distribution of SNP variants in the genomic region.

FIGURE S5 Dendrogram of 40 genotypes based on 39,137 SNPs.

TABLE S1 Weather data of three environments of the study.

TABLE S2 Descriptive statistic values of nine traits across three environments along with combined values for 40 genotypes.

TABLE S3 Environment wise correlation analysis of nine traits among 40 genotypes.

TABLE S4 Analysis of variance of nine traits of 40 genotypes across three environments.

TABLE S5 Alignment statistics of ddRAD reads of 40 genotypes.

TABLE S6 Genotype wise polymorphic SNPs (Read Depth = 10).

TABLE S7 Pair wise polymorphic SNPs between the genotypes.

TABLE S8 Transisionsvstransversions in the SNP variants of the study.

TABLE S9 Distribution of SNP variants across the chromosome in the genomic regions.

TABLE S10 Phenotypic values of genotypic groups.

TABLE S11 Details of associated SNPs with traits and their genomic positions.

TABLE S12 Genes in the proximate regions of associated SNPs with ZPR and SPY.
