# Supplementary material for: Stable SNP Allele Associations With High Grain Zinc Content in Polished Rice (Oryza sativa L.) Identified Based on ddRAD Sequencing
Source: Front Genet. 2020 Aug 11;11:763. doi: 10.3389/fgene.2020.00763 (PMC7432318; doi:10.3389/fgene.2020.00763)

Supplementary Figures

**Supplementary Figure 1A.** Morphological variation of seed of 40 genotypes in the study

**
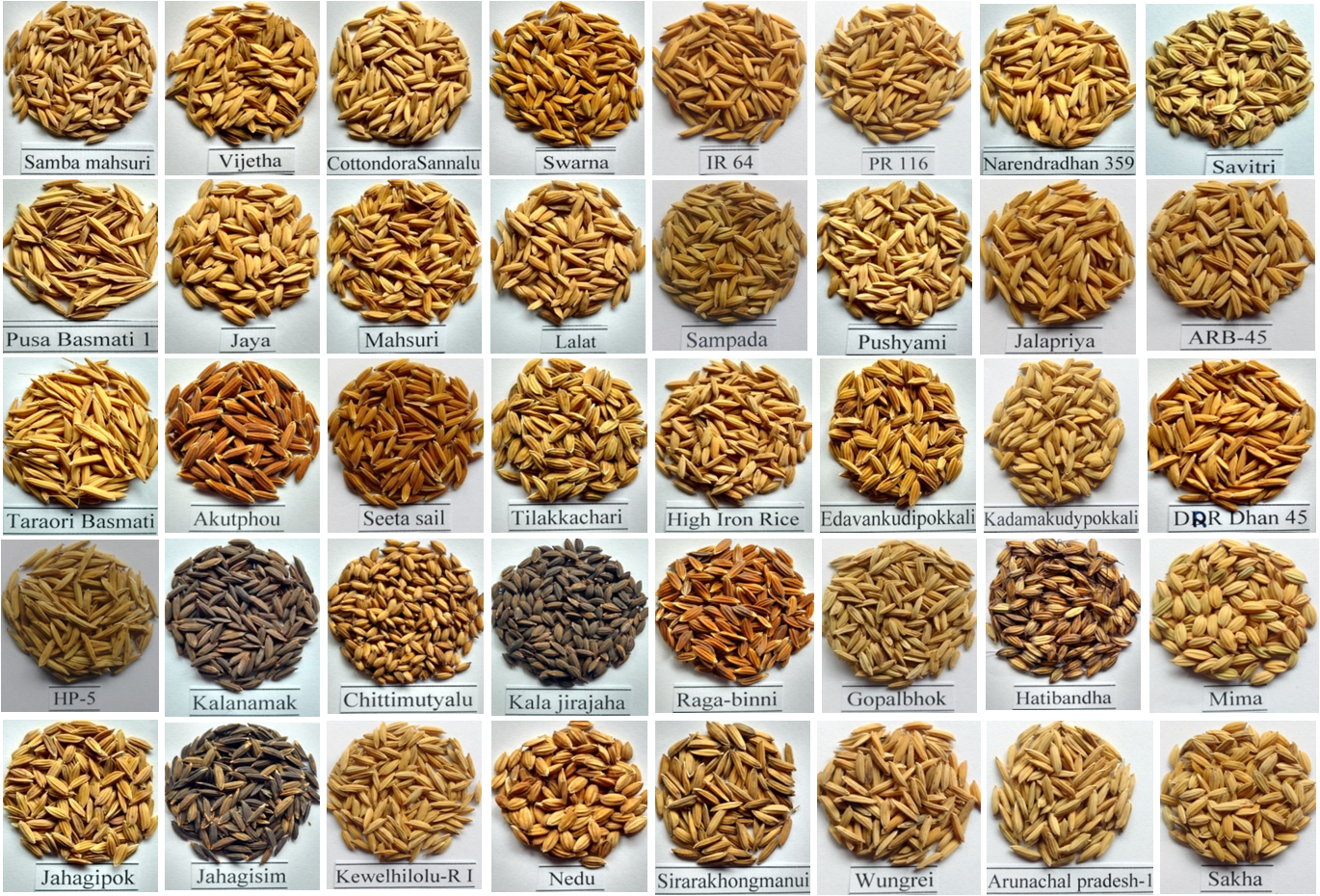
**

**Supplementary Figure 1B.** Frequency distribution plots for DFF-Days to fifty percent flowering; PH- Plant Height(cm); PL- Panicle Length(cm); NT- Number of Tillers per plant and SPY- Single Plant Yield (g).

**Supplementary Figure 1C.** Frequency distribution plots for IBR-Iron content in Brown Rice (ppm); ZBR- Zinc content in Brown Rice (ppm); IPR- Iron content in Polished Rice (ppm); ZPR- Zinc content in Polished Rice (ppm).

**Supplementary Figure 2.** Cluster dendrogram of 40 genotypes based on nine traits (DFF-Days to fifty percent flowering; PH- Plant Height (cm); PL- Panicle Length (cm); TNT- Total Number of Tillers per plant; SPY- Single Plant Yield (g); IBR-Fe content in Brown Rice (ppm); ZBR- Zn content in Brown Rice (ppm); IPR- Fe content in Polished Rice (ppm); ZPR- Zn content in Polished Rice (ppm)


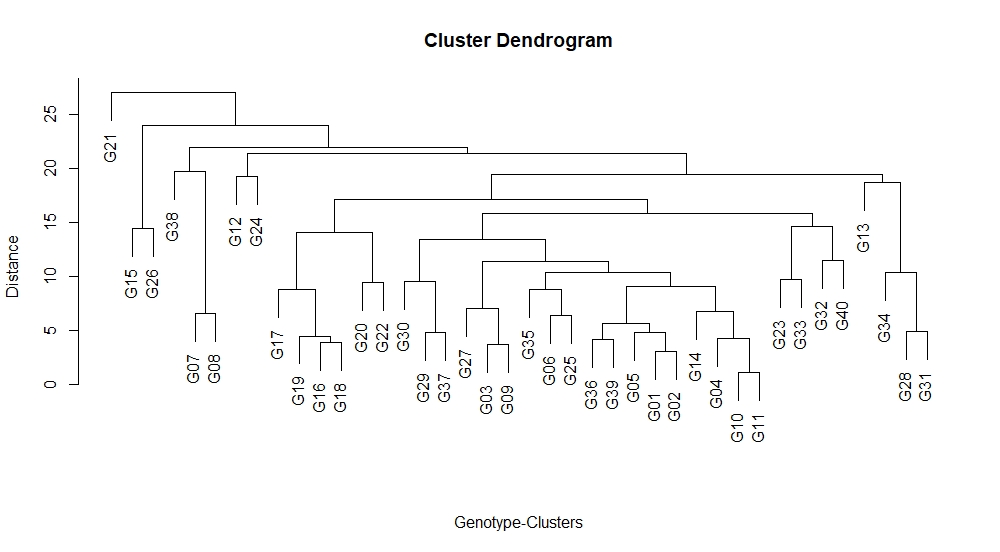


**Supplementary Figure 3.** Chromosome wise total SNPs of 40 genotypes at Read Depth 10.

**
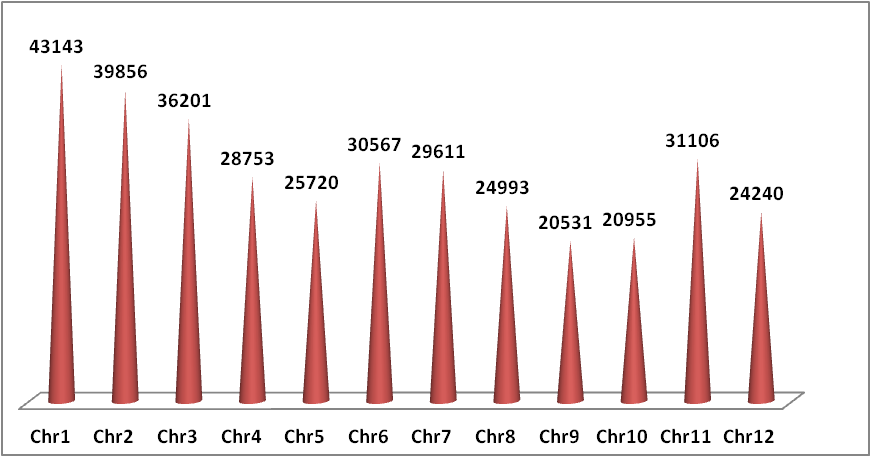
**

**Supplementary Figure 4.** Distribution of SNP variants in the genomic region

**
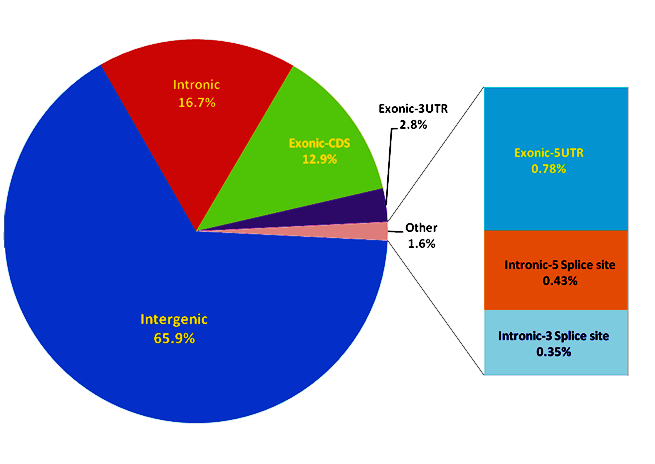
**

**Supplementary Figure 5.** Dendrogram of 40 genotypes based on 39,137 SNPs


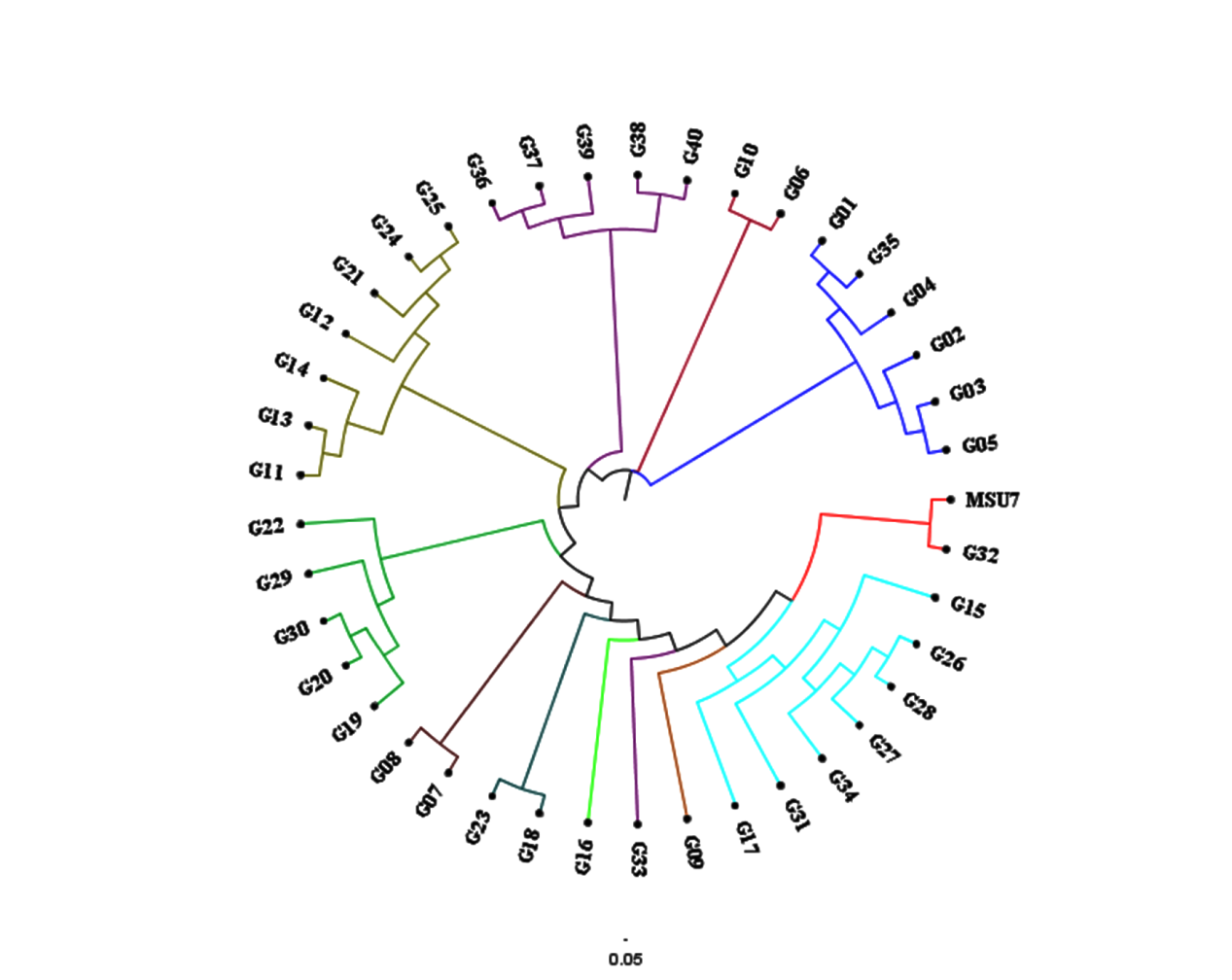

Supplement: FIGURE S1A — Morphological variation of seed of 40 genotypes in the study. [file Table_13.docx]
